# Early unplanned readmission of neurosurgical patients after treatment of intracranial lesions: a comparison between surgical and non-surgical intervention group

**DOI:** 10.1007/s00701-020-04521-4

**Published:** 2020-08-15

**Authors:** Caroline Sander, Henry Oppermann, Ulf Nestler, Katharina Sander, Nikolaus von Dercks, Jürgen Meixensberger

**Affiliations:** 1 Department of Neurosurgery, University Hospital Leipzig, Liebigstrasse 20, 04103 Leipzig, Germany; 2 Berlin, Germany; 3 Department for Medical Controlling, University Hospital Leipzig, Liebigstrasse 18, 04103 Leipzig, Germany

**Keywords:** Neurosurgery, Surgical treatment, Conservative treatment, Intervention, 30-day readmission, Unplanned readmission, Adverse events

## Abstract

**Background:**

Recent health care policy making has highlighted the necessity for understanding factors that influence readmission. To elucidate the rate, reason, and predictors of readmissions in neurosurgical patients, we analyzed unscheduled readmissions to our neurosurgical department after treatment for cranial or cerebral lesions.

**Methods:**

From 2015 to 2017, all adult patients who had been discharged from our Department of Neurosurgery and were readmitted within 30 days were included into the study cohort. The patients were divided into a surgical and a non-surgical group. The main outcome measure was unplanned inpatient admission within 30 days of discharge.

**Results:**

During the observation period, 183 (7.4%) of 2486 patients had to be readmitted unexpectedly within 30 days after discharge. The main readmission causes were surgical site infection (34.4 %) and seizure (16.4%) in the surgical group, compared to natural progression of the original diagnosis (38.2%) in the non-surgical group. Most important predictors for an unplanned readmission were younger age, presence of malignoma (OR: 2.44), and presence of cardiovascular side diagnoses in the surgical group. In the non-surgical group, predictors were length of stay (OR: 1.07) and the need for intensive care (OR: 5.79).

**Conclusions:**

We demonstrated that reasons for readmission vary between operated and non-operated patients and are preventable in large numbers. In addition, we identified treatment-related partly modifiable factors as predictors of unplanned readmission in the non-surgical group, while unmodifiable patient-related factors predominated in the surgical group. Further patient-related risk adjustment models are needed to establish an individualized preventive strategy in order to reduce unplanned readmissions.

**Electronic supplementary material:**

The online version of this article (10.1007/s00701-020-04521-4) contains supplementary material, which is available to authorized users.

## Introduction

Early readmission has emerged as a surrogate marker for assessing the quality of hospitals. Since financial hospital reimbursement policies are becoming more important, identifying risk factors for unplanned readmissions is of crucial interest [2, 24].

Mastering of reasons and predictive factors for unplanned readmission is useful for saving costs and optimizing resources. In addition to, and from a medical point of view far more important than economic reasons, the focus lies on defining patient groups at risk, to help in the development of preventive strategies and to increase patient safety and satisfaction [1, 6, 12]. Thirty-day hospital readmission is a marker associated to short-term complications and is often employed by health care politicians for outcome measurements [3].

In the literature, early re-hospitalization within 30 days after a neurosurgical procedure prognosticates an adverse outcome in patients with glioblastoma [5].

However, for neurosurgical procedures, there is a certain lack of readmission data. Especially readmission rates, predictive factors for readmission and outcome measurements after cranial neurosurgical treatment are missing. Due to the complex spectrum of neurosurgical diagnoses and the large scope in techniques and decision making, the already established predictive factors from other surgical or medical disciplines cannot be implemented unequivocally.

Patients with neurosurgical diseases suffer from a particular potential for rapid deterioration. Specific strategies and definition of risk factors to prevent unplanned readmission for this patient cohort are needed [22]. The incomparability of the different health systems worldwide is additionally complicating this procedure in neurosurgical conditions [8, 20]. Several datasets of neurosurgical patients from the USA are available [5, 13, 14, 22], but there is only few information about readmission analysis in European and especially the German health care system [19, 20, 23]. In addition, with the growing availability of interventional treatment and the aging population, the amount of patients without surgical treatment, meaning conservative or interventional neurosurgical therapy, is increasing. This patient group has been neglected in other readmission analyses and risk assessments.

The primary goal of this study was to identify predictive factors for unplanned early readmission in surgical and non-surgical groups in order to define patients with high risk. Additionally, we aimed to detect the causes of preventable readmissions as an approach to reduce readmission rates and to enable prevention strategies.

## Methods

The internal review board of the Medical Faculty of the University of Leipzig had agreed to the retrospective data analysis (167/18-ek). According to the approval of the ethics committee, the patient’s written consent is not required.

Assessing administrative data from January, 1^st^, 2015 to December, 31^st^, 2017, adult patients (> 18 years) who had undergone neurosurgical treatment for cranial or cerebral disease at the neurosurgical department were included in the monocentric, retrospective study. Index diagnoses were categorized according to the Classification of Diseases and Related Health Problems (10th Revision, German Modification; see Table S1 in supporting information).

From this group, patients were identified who were readmitted to any department or service at the University Hospital Leipzig within 30 days. We did not track readmissions to other hospitals. We excluded patients who came back for scheduled interventions. The first “index admission” diagnoses contain all cranial or cerebral neurosurgical disorders, irrespective of the following surgical or nonsurgical/interventional treatment. For the observation period, we reviewed the hospital charts of each readmitted patient and obtained demographic information.

Patient clinical complexity level (PCCL) is defined via the effective assessment ratio of the German DRG (diagnose related group) coding level and integrates also clinical course, technical procedures, and the patient’s secondary diagnoses. The PCCL represents a standardized measure of case severity; higher values indicate a higher case severity.

Causes for readmission were categorized into (1) surgical complications (e.g., surgical site infections (SSI), cerebrospinal fluid (CSF) leak, hemorrhage), (2) medical complications (e.g., electrolyte disorder, nosocomial infections, medication adverse effects), (3) diagnosis-related complications (e.g., tumor progression, hydrocephalus), (4) neurological decompensation (e.g., stroke, seizure, progressive neurologic symptoms), (5) pain management, and (6) miscellaneous (e.g., psychiatric admissions, “social” admissions due to lacking home care).

For addressing the wide variability and the complexity of neurosurgical disorders, we subcategorized the patients into groups with surgical and non-surgical/interventional treatment and compared the outcome separately. Four categories of readmission were defined: (1) preventable reasons (e.g., SSI, CSF leak, postoperative hemorrhage, nosocomial infection, postoperative pain, falls), (2) reasons related to the natural progression of the disease (e.g., hydrocephalus, new onset of seizures due to recurrent tumor growth), (3) reasons despite best practice (e. g., stroke, new neurological symptoms), and (4) unrelated reasons according to the study by Shah et al. [22]

## Statistical methods

Statistical analysis was performed with IBM SPSS Statistics 25.0 software (IBM, Armonk, New York, USA). The associations between continuous variables were examined using the *t* test, categorical variables employing the Fisher exact test. Continuous variables were described using mean values, while categorical variables were described with counts and frequencies. Binary multivariate logistic regression was used to assess significant predictive factors. Factors associated with an unplanned readmission at the univariate level with a *p* value of 0.20 or lower were integrated into the model. The threshold of continuous variables was estimated using the area under the curve. A two-tailed *p* value < 0.05 was considered to be statistically significant.

## Results

### Study population

The demographic and descriptive parameters are shown in Table 1. Overall, 2486 patients had been treated in the department of neurosurgery between 2015 and 2017. Most common index diagnoses for hospital admission were intracranial neoplasm (903 cases, 36.3%), followed by vascular diseases (834 cases, 33.6%) and traumatic injuries (408 cases, 16.4%). Altogether, 1412 patients (56.8 %) received a neurosurgical operation, whereas 1074 cases (43.2%) were treated non-surgically/interventionally. A total of 212 cases (15%) underwent more than one operation at index admission.

**Table 1:** Patient demographics and hospital characteristics of the study population

Whole group *n* = 2486		Average/No. (%)
Index admission diagnosis	Hydrocephalus	242 (9.74)
	Functional disorders	87 (3.50)
	Neoplasm	903 (36.32)
	Other	12 (0.48)
	Traumatic head injury	408 (16.41)
	Vascular disease	834 (33.55)
PCCL		2.87
Age in years		60,19
Age in years	> 65	1072 (43.12)
Discharge	Home	2166 (87.13)
	Rehabilitation	164 (6.60)
	External hospital	102 (4.10)
	Patientsʼ discretion	15 (0.60)
	Death	38 (1.53)
Gender	Female	1211 (48.71)
LOS in days		9.60
LOS in days	1–8	1468 (56.77)
	9–16	646 (24.98)
	17–24	240 (9.28)
	≥ 25	132 (5.31)
Treatment at ICU unit at index admission		503 (20.23)
Number transfer to ICU at index admission	1	450 (17.40)
	2	44 (1.70)
	≥ 3	9 (0.36)
LOS ICU at index admission in days		2.67
Surgical treatment		1412 (56.80)
Number surgeries per case		0.69
Number surgeries	1	1200 (46.40)
	2	150 (5.80)
	3	45 (1.74)
	≥ 4	17 (0.68)
Surgery time in minutes		146
Time point surgery^a^	Day shift	1224 (49.24)
Number second diagnoses per case		6.36
Comorbidity^b^		1317 (52.98)
Hypertension		1305 (52.49)
Cerebrovascular disease		695 (27.96)
Malignoma		426 (17.14)
Diabetes mellitus		359 (14.44)
Cardiovascular disease		423 (17.02)
Other^c^		182 (7.32)
Chronic kidney disease		149 (5.99)
Chronic obstructive pulmonary disease		123 (4.95)

*HIV* human immunodeficiency virus, *ICU* intensive care unit, *LOS* length of stay, *No.* number, *PCCL* patient clinical complexity level

^a^Surgery time: day time: 7 a.m. until before 7 p.m.; night shift: 7 p.m. until before 7 a.m.

^b^Comorbidity: yes in the case of three or more side diagnoses

^c^Other side diagnosis: infection with HIV; previous organ transplantation, oral anticoagulation, adipositas, malnutrition

The surgical study group showed a significantly higher case severity measured by PCCL, longer length of stay (LOS), and increased number of second diagnoses in comparison to the non-surgical/interventional group. In addition, patients after surgery were significantly more often discharged directly to rehabilitation, whereas patients in the non-surgical cohort were significantly more often discharged home or according to their discretion. Results are illustrated in Table 2.

**Table 2:** Patient demographics and hospital characteristics of the study population comparing non-surgical and surgical treatment. *p* values calculated by *t* test (mean values) or by Fisher exact test (frequencies)

Complete study population *n* = 2486		Non-surgical group *n* = 1074	Surgery group *n* = 1412	*p* value
		Average/No. (%)	Average/No. (%)	
Index admission diagnosis	Hydrocephalus	64 (5.96)	178 (12.61)	*0.0001*
	Functional disorders	7 (0.65)	80 (5.67)	*0.0001*
	Neoplasm	243 (22.63)	660 (46.74)	*0.0001*
	Other	5 (0.47)	7 (0.50)	1.000
	Traumatic head injury	285 (26.54)	123 (8.71)	*0.0001*
	Vascular disease	470 (43.76)	364 (25.78)	*0.0001*
Readmission rate		55 (5.12)	128 (9.07)	*0.0002*
Unplanned operation rate ^a^		10 (0.93)	68 (4.82)	*0.0001*
PCCL, index admission		1.48	3.3	*< 0.0001*
Discharge	Home	960 (89.39)	1207 (85.48)	*0.0044*
	Rehabilitation	47 (4.38)	117 (8.29)	*0.0001*
	Patientsʼ discretion	14 (1.30)	1 (0.07)	*0.0001*
Gender	Female	568 (52.89)	643 (45.54)	*0.0003*
LOS in days		5.88	12.42	*< 0.0001*
LOS in days	1–8	838 (78.03)	630 (44.62)	*0.0001*
	9–16	171 (15.92)	475 (33.64)	*0.0001*
	17–24	48 (4.47)	192 (13.60)	*0.0001*
	≥ 25–32	17 (1.58)	115 (8.14)	*0.0001*
Treatment at ICU		61 (5.69)	442 (31.30)	*0.0001*
Number transfer to ICU	1	59 (96.72)	391 (88.46)	*0.0001*
	2	2 (3.28)	42 (9.50)	*0.0001*
Number second diagnoses per case		6.11	6.55	*0.045*
Malignoma		150 (13.99)	276 (19.55)	*0.0003*
Cerebrovascular disease		325 (30.32)	370 (26.20)	*0.0271*
Oral anticoagulation		47 (4.38)	26 (1.84)	*0.0003*

In italics corresponds to a *p* value < 0.05

*ICU* intensive care unit, *LOS* length of stay, *No.* number, *PCCL* patient clinical complexity level

^a^Unplanned operation rate: unplanned readmission and operation

### Readmission cohort

Among the 2486 neurosurgical patients of the study population, 183 (7.4%) patients were readmitted for unplanned reasons (Tables 3 and 4). In readmitted patients, the most common diagnosis at index admission had been intracranial neoplasm (83 patients, 45.4%), among them most often glioblastoma (33 cases, 18% of readmitted cases). The second most common readmission diagnosis was vascular disease with 48 cases (26.2%).

**Table 3:** Patient demographics and hospital characteristics of the study population comparing with and without unplanned readmission. *p* values calculated by *t* test (mean values) or by Fisher exact test (frequencies)

Complete study population *n* = 2486		Without readmission *n* = 2303	Unplanned readmission *n* = 183	*p* value
		Average/No. (%)	Average/No. (%)	
Index admission diagnosis	Hydrocephalus	222 (9.64)	20 (10.93)	0.6035
	Functional disorders	80 (3.47)	7 (3.83)	0.8332
	Neoplasm	820 (35.61)	83 (45.36)	*0.0104*
	Other	12 (0.52)	0 (0.00)	1.000
	Traumatic head injury	383 (16.63)	25 (13.66)	0.3504
	Vascular disease	786 (34.13)	48 (26.23)	*0.0342*
PCCL		2.85	3.07	0.323
Age in years		60.40	57.65	0.053
Gender	Female	1134 (49.24)	77 (42.08)	0.0653
LOS in days		9.39	12.24	*< 0.0001*
LOS in days	1–8	1395 (60.57)	73 (39.89)	*0.0001*
	9–16	580 (25.18)	66 (36.07)	*0.0021*
	17–24	210 (9.12)	30 (16.39)	*0.0026*
	≥ 25–32	118 (5.12)	15 (8.20)	0.0861
Treatment at ICU at index admission		456 (19.80)	47 (25.68)	0.0687
Number transfer to ICU	1	408 (89.47)	42 (89.36)	1.000
	2	39 (8.55)	5 (10.64)	0.5890
	≥ 3	9 (0.39)	0 (0.00)	1.000
Surgical treatment at index admission		1284 (55.75)	128 (69.95)	*0.001*
Number surgeries per case		0.67	0.896	*0.0001*
Number second diagnoses per case		6.30	7.20	*0.013*
Comorbidity^a^		1196 (51.93)	121 (66.12)	*0.0002*
All cardiac diseases^b^		652 (28.31)	67 (36.61)	*0.0219*
Malignoma		367 (15.94)	59 (32.24)	*0.0001*
Oral anticoagulation		69 (3.00)	4 (2.19)	0.8184

In italics corresponds to a *p* value < 0.05

*ICU* intensive care unit, *LOS* length of stay, *No.* number, *PCCL* patient clinical complexity level

^a^comorbidity: yes in the case of three or more side diagnoses

^b^All cardiac diseases: ischemic heart diseases, pulmonic heart diseases, other heart diseases, cardiovascular diseases, cardiovascular devices

**Table 4:** Patient demographics and hospital characteristics of the unplanned readmitted study population

Unplanned readmission cohort *n* = 183		Average/No. (%)
Unplanned readmission		183 (100)
Readmission rate (of 2486)		7.36
Unplanned operation^a^ (of 183)		78 (42.62)
Unplanned operation rate to whole cohort^b^ (of 2486)		3.14
LOS at readmission in days		9.81
Average number readmission per case		1.15
Average number readmission	1	140 (76.50)
	≥ 2	18 (23.50)
Time from index surgery to readmission in days		20.55
Surgery time in minutes at index admission		152
Time point, index surgery^c^	Night shift	13 (7.10)
Time from discharge to readmission in days		10.95
Readmission cause	Surgical	76 (41.53)
	Medical	20 (10.93)
	Original diagnosis	19 (10.38)
	Neurological decompensation	34 (18.58)
	Pain management	14 (7.65)
	Miscellaneous	20 (10.93)
Surgical site infection	Superficial	12 (6.56)
	Deep	22 (12.022)
	CSF leak	12 (6.56)
	Shunt infection	3 (1.64)
Readmission category	Preventable	79 (43.17)
	Despite best practice	46 (25.14)
	Natural progression of the disease	47 (25.68)
	Unrelated	11 (6.01)

*CSF* cerebrospinal fluid, *LOS* length of stay, *No.* number

^a^Unplanned operation: unplanned readmission and operation

^b^Unplanned operation rate: unplanned readmission and operation compared to whole cohort

^c^Surgery time: day time: 7 a.m. until before 7 p.m., night shift: 7 p.m. until before 7 a.m.

The majority of patients was readmitted to our neurosurgical department (80.3%), followed by readmission to the operative intensive care unit (9.3%) and the department of internal medicine (6% with 2.2% readmission to the internal intensive care unit).

The most frequent causes of readmission were surgery-related adverse events, followed by neurological deterioration and by difficulties in pain management. Of the 76 patients (41.5%) readmitted for surgical complications, 64.5% were readmitted for SSI, 22.7% for recurrent hemorrhage, and 6.7% for shunt dysfunction. Of the 34 patients admitted due to neurological deterioration, 76.5% had a seizure and 5.9% developed hydrocephalus. Besides infections (33.3%) and seizures (14.2%), unspecific reasons (19.1%) were among the most frequent causes for unplanned readmission. They mostly consisted in deterioration of the patient’s general condition. Unplanned operations were needed in 78 cases, the major reasons were SSI (30 cases; 38.5%), recurrent bleeding (17 cases; 21.8%), and shunt dysfunction (4 cases; 5.1%).

### Preventable readmission

The reasons for readmission were carefully analyzed based on the previously described criteria to determine whether a readmission might have been prevented [22]. Among the 183 unplanned readmissions, 79 cases (43.2%) were classified as preventable (Table 4). The majority of preventable readmissions were caused by SSI, postoperative complications, or postoperative pain.

### Procedure-related readmission

The assessment of the 183 readmitted patients stratified to the treatment modality at first index admission included 55 patients with non-surgical treatment and 128 patients with surgery (Table 5). Comparison confirmed significant differences in most examined aspects, especially the PCCL and LOS. Readmission cause was significantly more often a surgical problem, and category set as “preventable” in the operated patients, but significantly less due to the “original diagnosis” and “other” reasons. “Other” readmission reasons in the non-surgical group were unspecific deterioration such as vertigo, nausea, or vomiting (8 cases).

**Table 5:** Patient demographics and hospital characteristics comparing unplannedly readmitted patients in the non-surgically and surgically treated groups. *p* values calculated by *t* test (mean values) or by Fisher exact test (frequencies)

Unplanned readmission *n* = 183		Non-surgical group *n* = 55 out of 1074	Surgery group *n* = 128 out of 1412	*p* value
		Average/No. (%)	Average/No. (%)	
Unplanned readmission (unplanned readmission rate)		55 (5.12)	128 (9.07)	*0.0002*
Unplanned operation		10 (18.18)	68 (53.13)	*0.0001*
PCCL, index admission		1.39	3.79	*< 0.0001*
Age in years	> 65	29 (52.73)	45 (35.16)	*0.0328*
LOS index admission in days		7.64	14.21	*< 0.0001*
LOS, index admission in days	1–8	38 (69.09)	35 (27.34)	*0.0001*
	9–16	10 (18.18)	56 (43.75)	*0.0013*
Treatment at ICU at index admission		7 (12.73)	40 (31.25)	*0.0095*
Number second diagnoses per case		7.25	7.18	*0.045*
Cardiovascular disease		18 (32.73)	23 (17.97)	0.0988
LOS at readmission in days		8.47	10.38	*0.004*
LOS at readmission in days	1–8	40 (72.73)	71 (55.47)	*0.0324*
	9–16	7 (12.73)	36 (28.13)	*0.0240*
Average number readmission	1	39 (78.00)	101 (92.66)	*0.0154*
	2	9 (18.00)	6 (5.50)	*0.0185*
Readmission cause	Surgical	12 (21.82)	64 (50.00)	*0.0005*
	Medical	7 (12.73)	13 (10.16)	0.6119
	Original diagnosis	11 (20.00)	8 (6.25)	*0.0080*
	Neurological decompensation	7 (12.73)	27 (21.09)	0.2174
	Pain management	7 (12.73)	7 (5.47)	0.1266
	Miscellaneous	11 (20.00)	9 (7.03)	*0.0177*
Surgical site infection	Superficial	3 (60.00)	9 (20.45)	*0.0432*
	Deep	1 (20.00)	21 (47.73)	0.6142
	CSF leak	1 (20.00)	11 (25.00)	1.000
	Shunt infection	0 (0.00)	3 (6.82)	1,000
Readmission category	Preventable	17 (30.91)	62 (48.44)	*0.0344*
	Despite best practice	10 (18.18)	36 (28.13)	0.1941
	Natural progression of the disease	21 (38.18)	26 (20.31)	*0.0160*
	Unrelated	7 (12.73)	4 (3.13)	*0.0185*

*CSF* cerebrospinal fluid, *ICU* intensive care unit, *LOS* length of stay, *No.* number, *PCCL* patient clinical complexity level

In the non-surgical group, “preventable” readmissions consisted of SSI, triggered by previous surgeries dating back more than 30 days before the index admission (five cases), insufficient pain therapy (seven cases), or repeated falls (three cases). The majority of unplanned readmissions were categorized as “natural progression”, including seizures (5 cases) and non-specific complaints such as progressive neurological deficits or nausea (11 cases).

In the group of operated patients (Table 6), surgical patients with unplanned readmission were significantly younger (< 65 years), suffered more often from comorbidities, and stayed longer at index admission than patients without the need for readmission. The frequencies of concurrent malignancies and pulmonic heart disease were significantly higher in readmitted surgical patients.

**Table 6:** Patient demographics and hospital characteristics in unplannedly readmitted patients and patients without unplanned readmission in the surgically treated group. *p* values calculated by *t*-test (mean values) or by Fisher exact test

		Unreadmitted surgery group *n* = 1284	Readmitted surgery group *n* = 128	*p* value
		Average/No. (%)	Average/No. (%)	
Age in years		60.50	55.91	*0.0029*
Age in years	> 65	571 (44.47)	45 (35.16)	*0.0494*
LOS index admission in days		12.24	14.21	*0.0258*
LOS in days	1–8	595 (46.34)	35 (27.34)	*0.0001*
Comorbidity ^a^		699 (54.44)	84 (65.63)	*0.0154*
Pulmonic heart disease		11 (0.86)	4 (3.13)	*0.0400*
Malignoma		232 (18.07)	44 (34.38)	*0.0001*

In italics corresponds to a *p* value < 0.05

*LOS* length of stay, *No.* number

^a^Comorbidity: yes in the case of three or more side diagnoses

With regard to the non-surgical group (Table 7), readmitted patients had had a significantly longer LOS at index admission and a higher frequency of ICU treatment. The unplanned readmitted cases in the non-surgical group significantly more often were diagnosed with pre-existing cardiac diseases or malignancies.

**Table 7:** Patient demographics and hospital characteristics in unplannedly readmitted patients and patients without unplanned readmission in the non-surgically treated group. *p* values calculated by t-test (mean values) or by Fisher exact test

		Unreadmitted non-surgical group *n* = 1019	Readmitted non-surgical group *n* = 55	*p* value
		Average/No. (%)	Average/No. (%)	
LOS in days		5.79	7.64	*0.0281*
Treatment at ICU at index admission		47 (4.61)	7 (12.73)	*0.0169*
Transfer to ICU	1	45 (95.74)	7 (100.00)	*0.0196*
	≥ 2	2 (4.26)	0 (0.00)	1.00
Other heart diseases		102 (10.01)	11 (20.00)	*0.0379*
Cardiovascular disease		141 (13.84)	14 (25.46)	*0.0277*
All cardiac diseases^*a*^		53 (5.20)	39 (70.91)	*0.0001*
Diabetes mellitus		134 (13.15)	13 (23.64)	*0.0411*
Malignoma		135 (13.25)	15 (27.27)	*0.0079*

In italics corresponds to a *p* value < 0.05

*ICU* intensive care unit, *LOS* length of stay, *No.* number

^a^All cardiac diseases: ischemic heart diseases, pulmonic heart diseases, other heart diseases, cardiovascular diseases, cardiovascular devices

### Predictors for unplanned readmission

We identified patient-dependent predictors for unplanned readmission in the study groups (Table 8). The side diagnosis “malignoma” was significantly more frequent in readmitted patients of both groups. While in the surgical group, the presence of secondary diseases was predictors of unplanned readmission, we identified the treatment-dependent factors LOS and the need for intensive care as predictors in the non-surgical group. It is worth mentioning that a lower patient age (under 78 years) in the surgical group was associated to predict unplanned readmission. In addition, low PCCL (under 1) was found to be predisposing for unplanned readmission in the non-surgical group, although this relationship did not become statistically significant.

**Table 8:** Predictors for unplanned readmission. Multivariate binary logistic regression analysis for unplanned readmission in the study population. Factors with a *p* value of 0.20 or lower at the univariate level were integrated into the model. Threshold of continuous variables was estimated using the area under the curve

Multivariate regression	OR (95% CI) for readmission	*p* value
Surgical group (*n* = 1412)		
Higher age	0.98 (0.970–0.995)	*0.005*
Age > 77 years	0.89 (0.423–1.858)	0.749
Higher number of second diagnoses	0.97 (0.923–1.018)	0.216
Number of second diagnoses > 3	2.79 (1.478–5.273)	*0.002*
Presence of cormobidity	1.16 (0.662–2.038)	0.602
Presence of pulmonic heart disease	3.73 (1.072–12.999)	*0.039*
Presence of malignoma	2.44 (1.592–3.724)	*< 0.0001*
Presence of cerebrovascular disease	1.73 (1.106–2.701)	*0.016*
Presence of cardial stents	1.32 (0.556–3.124)	0.530
Non-surgical group (*n* = 1074)		
Gender, male	1.35 (0.760–2.407)	0.305
Higher PCCL	0.78 (0.578–1.054)	0.106
PCCL > 1	0.66 (0.295–1.366)	0.245
Longer LOS	1.07 (1.001–1.14)	*0.046*
LOS > 5 days	1.59 (0.696–3.629)	0.271
Treatment at ICU	5.79 (2.109–15.889)	*0.001*
Higher number of second diagnoses	0.95 (0.879–1.024)	0.178
Number second diagnoses > 3	2.63 (0.963–7.180)	0.059
Presence of comorbiditis	1.17 (0.453–3.020)	0.746
Presence of ischemic heart disease	3.09 (0.754–12.638)	0.117
Presence of other heart diseases	4.70 (0.832–26.566)	0.080
Presence of cardiovascular diseases	2.55 (0.399–16.222)	0.323
Presence of diabetes mellitus	1.64 (0.784–3.449)	0.188
Presence of malignoma	2.53 (1.287–4.979)	*0.007*
Presence of chronic obstructive lung disease	2.08 (0.759–5.715)	0.155
Previous organ transplantation	22.15 (1.209–405.746)	*0.037*

In italics corresponds to a *p* value < 0.05

*CI* 95% confidence interval, *LOS* length of stay, *ICU* intensive care unit, *OR* odds ratio, *PCCL* patient clinical complexity level

^a^Comorbidity: yes in the case of three or more side diagnoses

## Discussion

### Placing our results into the literature context

To the best of our knowledge, the present analysis constitutes the first attempt to address predictive factors for early readmission after treatment of intracranial lesions and to distinguish between surgical and non-surgical/interventional treatment.

Nearly half of the reasons for unplanned readmission were categorized as preventable (43.2%) and are mainly due to SSI. Looking at the surgical group, we found a readmission rate of 9.1% and a SSI rate of 34.4%. Similar rates of preventable readmissions (36.6%) have been published in neurosurgical patients [22].

In a comparable cohort of neurosurgical patients from Germany, a SSI rate of 4.1% in total and of 22.4% for unplanned readmitted patients was shown [19, 23]. Further studies from other disciplines reported SSI rated between 0.5% and 6.6% [10, 11, 16, 21]. However, it must be taken into account that neurosurgical patients are more readily hospitalized than other patients, because of the special nature of wound infections with their proximity to the central nervous system and the associated increased risk of complications, even in the case of superficial infections [22].

The second leading cause of readmission in our collective was new onset of seizures (14.2% of all readmissions, 1.9% of the complete population). Due to the nature of this adverse event, we categorized seizures as “readmission despite best practice”. A somewhat lower rate of newly occurring seizures was described in the literature with 3.69% of readmitted patients [22]. Slight differences regarding the use of categorization classes (“preventable” versus “best practice”) can explain the high seizure rate in the present study.

In our cohort, we found a nosocomial infection rate of 2.2%, lower than in comparable publications (12.1%) [19]. Furthermore, venous thrombotic events were described as a major cause for preventable readmission [5], which were completely absent in our study. A potential explanation may be the high specialization of neurosurgical departments with wide catchment areas. Consequently, patients with thromboembolism are eventually readmitted to non-neurosurgical departments of other hospitals to treat thrombosis, close to the residence of the patients.

### Procedure-related readmission

Many readmission-associated factors can be explained by the previous surgery and the increased risk of intervention-related complications with an extended LOS. Prolonged LOS points to the presence of adverse events and to an increased complexity of the case, which both in consequence can lead to secondary unplanned readmission [19]. In addition to unplanned reoperations, seizures, catheter-associated infections, or thromboembolic events are risk factors related to surgery, which non-operated patients are less likely to encounter.

In our study, the unplanned operation rate was with 4.8% significantly higher in the surgery group than in the non-surgical group (0.9%). The indications for unplanned surgery in the non-surgical group occurred, when enlarging or recurring subdural hematoma, as well as SSI in a first attempt had been treated conservatively, but then needed operation after further observation. Very similar to our results, unplanned operation rates of 3.1% due to postoperative bleeding, SSI, re-resection of a tumor, or CSF leakage have been published [4].

Readmission was significantly more often categorized as preventable in the surgical group than in the non-surgical group.

The unplanned readmissions in the non-surgical group were mainly due to unspecific complaints such as neurological deterioration and vertigo belonging to the category “natural progression”. In addition, significantly more patients from the non-surgical group were readmitted multiple times. We suppose that the unplanned readmissions in this group are mainly due to home care and nursing problems.

The readmitted patients in the non-surgical group are significantly older (> 65 years) and suffer from more secondary diagnoses than the patients in the surgical group, whereas the PCCL score of severity lies significantly lower (Table 5). It must be taken into account that the PCCL is a mixed value between the severity of the patient’s previous illnesses and the severity of the inpatient stay, whereby invasive procedures or complications have a higher influence. This mirrors that the unplanned readmissions in the operated group are due to a majority of young, mainly male patients after severe traumatic brain injury, as has been supposed before [18].

Our study shows that patients with previous cardiac diseases in particular were more likely to be unplannedly readmitted. This was striking in both study groups (Tables 6 and 7). For the non-surgical group, patients with diabetes mellitus and malignoma as secondary diagnoses were significantly more frequently readmitted. On the one hand, due to the higher patient age and on the other hand due to the abundance of secondary diseases, this is why non-surgical treatment may have been favored at the index admission.

### Non-modifiable and modifiable risk factors for an unplanned readmission

Main predictors for an unplanned 30-day readmission in the surgical group were a higher number of side diagnoses (> 3), presence of certain secondary diseases, and patient age, while the presence of a malignant tumor as side diagnosis showed a highly significant impact in both study groups. This is in good accordance with most comparable studies [7, 13, 15]. Other second diagnoses with implications on readmission are cerebral metastasis, congestive heart failure, peripheral arterial disease [15, 19], myocardial infarction [13], hypertension [4], or coagulopathy [15]. In the literature, the number of side diagnoses correlates significantly with an increased risk of 30-day reoperations, readmissions, mortality, and infections [20], similar to the results presented here.

In the present study population, further reported patient characteristics such as male gender [4], type of health insurance [7], income [17], or race [15] were not confirmed. In the non-surgical group, we identified mainly treatment-related factors to be predictive for 30-day readmission, such as longer LOS and the need for ICU treatment.

A longer LOS was predictive for unplanned readmission after neurosurgical treatment in the non-surgical cohort. A previous study had found that a longer LOS is associated with an increased likelihood of unplanned readmission [9].

Treatment-dependent factors (LOS, ICU) predicted unplanned re-hospitalization in non-operated patients, whereas patient-dependent factors (age and second diagnoses) dominated in the surgical group. Knowledge about modifiable predictors for unplanned readmission in non-surgical patients is new and essential for early identification and protection of patients with increased risk. It remains to be elucidated, in how far the surgical indication, the LOS, and the intensive care unit treatment are modifiable factors subjected to medical decisions, or if they rather constitute indicators for a complex clinical course, entailing more severe sequelae and a higher risk for 30-day readmission.

Identification of risk factors for 30-day readmission in these conservatively treated patients is of clinical relevance, not only to know the baseline readmission rate the neurosurgeon has to compete with, but also to be aware of factors putting the patient at risk already at the time of first patient counseling.

Strategies for reducing preventable unplanned readmission due to modifiable factors are the subject of current research. A modification of care upon discharge and post-discharge follow-up is being discussed [5]. Similar to the results described by Marcus et al., we determined a high frequency of unplanned readmissions caused by seizures and surgical site infections. A strict anticonvulsive prophylaxis and continued antibiotic regimes can help to reduce readmission rates [13]. A detailed discharge bundle and frequent follow-up appointments may reduce readmission rates and improve outcome.

### Limitations

Among the limitations of the study are the retrospective character and the data collection based on hospital documentation and coding systems. In addition, only patients who were re-hospitalized to the Leipzig University Hospital could be tracked. Even though 183 readmitted patients were identified, the numbers of patients become very small after subdivision, thus lowering the statistical power. The classification of different diseases into five diagnosis groups (Table S1) conceals certain important aspects, e.g., tumor entities or acute versus chronic hemorrhages. To address this, our ongoing research deals with detailed investigation of subentities and further reports are in preparation.

## Conclusions

The present study provides a comprehensive look at 30-day all-cause readmissions after neurosurgical treatment. Factors associated with an increased readmission rate are a younger patient age (< 65 years), longer LOS, and comorbidities. Patients who have undergone surgery have to be readmitted more frequently, often due to surgical site infection or seizures. Non-surgically or interventionally treated patients are most likely to be readmitted with neurological deterioration and unspecific complaints due to natural progression of the index diagnosis.

In operated patients, main predictors for readmission were non-modifiable, such as age and comorbidities, whereas in non-surgically treated patients, the seemingly modifiable predictors LOS and intensive care unit treatment were identified. In both groups, the presence of a malignant disease as side diagnosis strongly predicted 30-day readmission. Patient counseling and information to relatives have to consider the non-modifiable factors at the beginning of the treatment, especially for neurosurgical decision making.

The analysis of readmission rates and established risk factors is the beginning of individualized risk adjustment and will help to form quality improvement programs in the future.

## Electronic supplementary material

ESM 1 (DOCX 15 kb)

## Data Availability

Data of this work is available from corresponding author upon reasonable request.

## Abbreviations

CSF: Cerebrospinal fluid
CI: Confidence interval
e.g.: Example given
HIV: Human immunodeficiency virus
ICU: Intensive care unit
ICD-10-GM: Classification of Diseases and Related Health Problems, 10th Revision, German Modification
LOS: Length of stay
n.a.: Not applicable
No.: Number
OR: Odds ratio
PCCL: Patient clinical complexity level
USA: United States of America
