# Supplementary material for: Early unplanned readmission of neurosurgical patients after treatment of intracranial lesions: a comparison between surgical and non-surgical intervention group
Source: Acta Neurochir (Wien). 2020 Aug 15;162(11):2647–58. doi: 10.1007/s00701-020-04521-4 (PMC7550291; doi:10.1007/s00701-020-04521-4)
Supplement: Supplementary file 1 — (DOCX 15 kb) [file 701_2020_4521_MOESM1_ESM.docx]

**Supporting information**

**S1 Table.** Included diagnoses for the five diagnosis groups according to ICD-10-GM

| diagnosis groups | |  |
| --- | --- | --- |
| neoplasm | C70 | malignant neoplasm of the meninges |
|  | C71 | malignant neoplasm of the brain |
|  | C72 | malignant neoplasm of the spinal cord, cranial nerves and other parts of the central nervous system |
|  | C79.4 | secondary malignant neoplasm of other and unspecified parts of the nervous system |
|  | C79.3 | secondary malignant neoplasm of the brain and meninges |
|  | C79.5 | secondary malignant neoplasm of the bone and bone marrow |
|  | D32 | benign neoplasm of meninges |
|  | D33 | benign neoplasm of the brain and other parts of the central nervous system |
|  | D35 | benign neoplasm of other and unspecified endocrine glands |
|  | D42 | neoplasm of uncertain or unknown behaviour of the meninges |
|  | D43 | neoplasm of uncertain or unknown behaviour of the brain and central nervous system |
|  | D44 | neoplasm of uncertain or unknown behaviour of endocrine glands |
|  | D47 | other neoplasms of uncertain or unknown behaviour of lymphatic, hematopoietic and related tissues |
| hydrocephalus | G91 | hydrocephalus |
|  | G93.0 | brain cysts |
|  | G93.2 | benign intracranial hypertension |
|  | Q03 | congenital hydrocephalus |
|  | Q07 | other congenital malformations of the nervous system |
| vascular | D18.02 | intracranial hemangioma |
|  | G45 | cerebral transitory ischemia and related syndromes |
|  | G46* | cerebral vascular syndromes in cerebrovascular diseases |
|  | I60 | subarachnoid hemorrhage |
|  | I61 | intracerebral hemorrhage |
|  | I62 | other non-traumatic intracranial bleeding |
|  | I63 | cerebral infarction |
|  | I64 | stroke, not called hemorrhage or infarction |
|  | I65 | occlusion and stenosis of precerebral arteries without resulting stroke |
|  | I66 | occlusion and stenosis of cerebral arteries without resulting cerebral infarction |
|  | I67 | other cerebrovascular diseases |
|  | I68* | cerebrovascular disorders in diseases classified elsewhere |
|  | I69 | consequences of a cerebrovascular disease |
|  | I72.0 | aneurysm and dissection of the carotid artery |
|  | I72.5 | aneurysm and dissection of other precerebral arteries |
|  | I72.6 | aneurysm and dissection of the vertebral artery |
|  | Q28.2 | arteriovenous malformation of the cerebral vessels |
|  | Q28.3 | other malformations of the cerebral vessels |
| trauma | S00 | superficial injury of the head |
|  | S01 | open wound of the head |
|  | S02 | fracture of the skull and craniofacial bones |
|  | S03 | luxation, sprain and strain of joints and ligaments of the head |
|  | S04 | cranial nerve injury |
|  | S05 | injury of the eye and the orbit |
|  | S06 | intracranial injury |
|  | S07 | crushing of the head |
|  | S08 | traumatic amputation of parts of the head |
|  | S09 | other and unspecified injuries of the head |
| functional | G10 | chorea Huntington |
|  | G20 | primary Parkinson syndrome |
|  | G21 | secondary Parkinson syndrome |
|  | G22* | Parkinson syndrome in diseases classified elsewhere |
|  | G24 | dystonia |
|  | G25 | other extrapyramidal diseases and movement disorders |
|  | G40 | epilepsy |
|  | G80 | infantile cerebral palsy |
